# Arthroscopic Saucerization of a Symptomatic Posterior Horn Tear in a Discoid Medial Meniscus

**DOI:** 10.31486/toj.19.0125

**Published:** 2021

**Authors:** Bhumit Desai, Michael Warren, Lacey G. Lavie, Michael Nammour, Sean Waldron

**Affiliations:** ^1^ The University of Queensland Faculty of Medicine, Ochsner Clinical School, New Orleans, LA; ^2^ Department of Orthopedic Surgery, Ochsner Clinic Foundation, New Orleans, LA

**Keywords:** *Arthroscopy*, *knee*, *meniscus*, *tibial meniscus injuries*

## Abstract

**Background:** Discoid medial meniscus is an extremely rare congenital anatomic variant with an estimated incidence of 0.12%. Arthroscopic meniscal saucerization and repair are reserved for symptomatic tears only. We present a case of discoid medial meniscus tear, outline the surgical arthroscopic technique used for treatment, and compare several surgical approaches.

**Case Report:** An 18-year-old male presented with left knee pain and mechanical symptoms present for 2 years. Physical examination showed stability to both varus and valgus stresses with absence of locking or catching on McMurray testing. Magnetic resonance imaging confirmed discoid medial meniscus with a horizontal oblique tear of the posterior horn. The patient underwent saucerization of the left discoid medial meniscus and medial meniscus repair.

**Conclusion:** Discoid medial meniscus predisposes individuals to meniscal tears that often require operative management. Careful consideration of surgical approach can help to optimize patient outcomes while minimizing the risk of iatrogenic injury.

## INTRODUCTION

The medial meniscus is a semicircular structure of fibrocartilage that covers up to 60% of the articular surface of the medial tibial plateau.^1^ The main loading occurs at the posterior region of the meniscus, with the posterior horn sliding over the posterior rim of the tibial plateau during knee flexion. Deep knee flexion puts significant stress on the posterior horn and represents a high-risk area for medial meniscal injury.^2^ Discoid medial meniscus is an extremely rare congenital anatomic variant that can be diagnosed in symptomatic pediatric meniscal tears. The estimated incidence of discoid medial meniscus is 0.12%; however, the true incidence is difficult to determine given the unknown percentage of asymptomatic discoid medial menisci.^3^ Clinical and radiographic evaluation is warranted in patients with symptomatic knee pain, effusion, and frank locking or catching.^3^ Trauma has been reported as the inciting cause of symptoms in up to 66% of patients.^3^ Surgical treatment is reserved for symptomatic patients because asymptomatic patients do not require prophylactic operative intervention.^4^

Cave and Staples described the first case of discoid medial meniscus in 1941.^5^ Although an extremely rare entity with fewer than 70 reported cases worldwide, discoid medial meniscus has been well characterized in the literature.^6-10^ Arthroscopic treatment techniques have also been described.^11^

Discoid lateral meniscus is another anatomic variant and is also well described in the literature, with an estimated incidence of up to 5% of the US population.^12^ Discoid lateral meniscus tends to manifest as knee hypermobility in childhood without any tear, whereas discoid medial meniscus is asymptomatic in childhood until injury or other inciting event. The management goals in discoid lateral meniscus tears are identical to those for discoid medial meniscus tears: meniscal reshaping through arthroscopic saucerization to facilitate the preservation of articular surfaces between the femur and tibia. Long-term follow-up is lacking, although results from arthroscopic saucerization of discoid lateral meniscus tears have been extrapolated to justify saucerization of symptomatic discoid medial meniscus tears.^13^ Despite the similar goals, the surgical approach and technique differ when treating discoid lateral meniscus vs discoid medial meniscus.

We present a case of discoid medial meniscus tear treated with arthroscopic saucerization and subsequent repair and examine different surgical approaches used to treat this pathology.

## CASE REPORT

An 18-year-old male presented to the pediatric orthopedic clinic for evaluation of left knee pain. Pain and subjective symptoms of locking and catching had started more than 2 years prior, causing the patient to have significant difficulty with activities of daily living. Continued symptoms of medial knee pain, locking, and instability led the patient to seek medical evaluation.The patient recalled no traumatic events.

**Figure 1.**
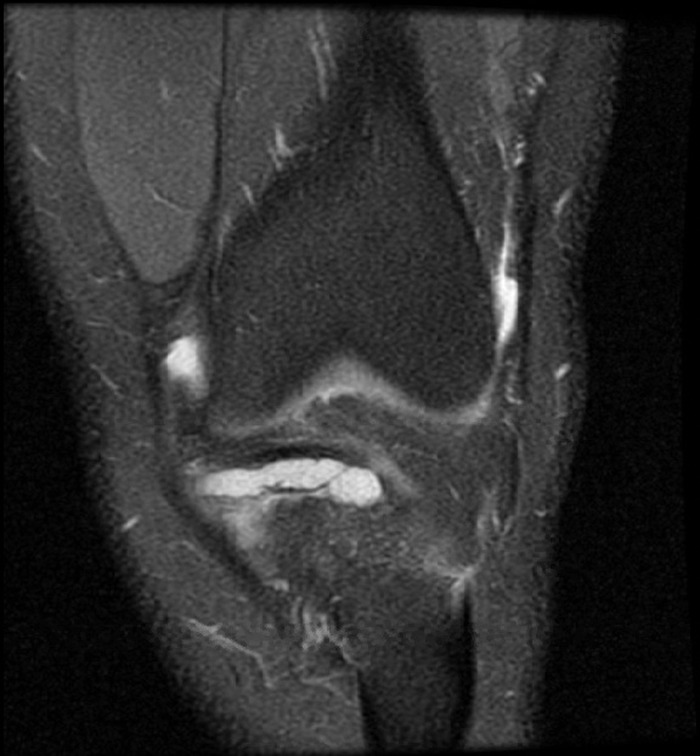
**Coronal proton density fat-suppressed magnetic resonance image shows a right discoid medial meniscus measuring 3.58 × 0.74 cm.**

**Figure 2.**
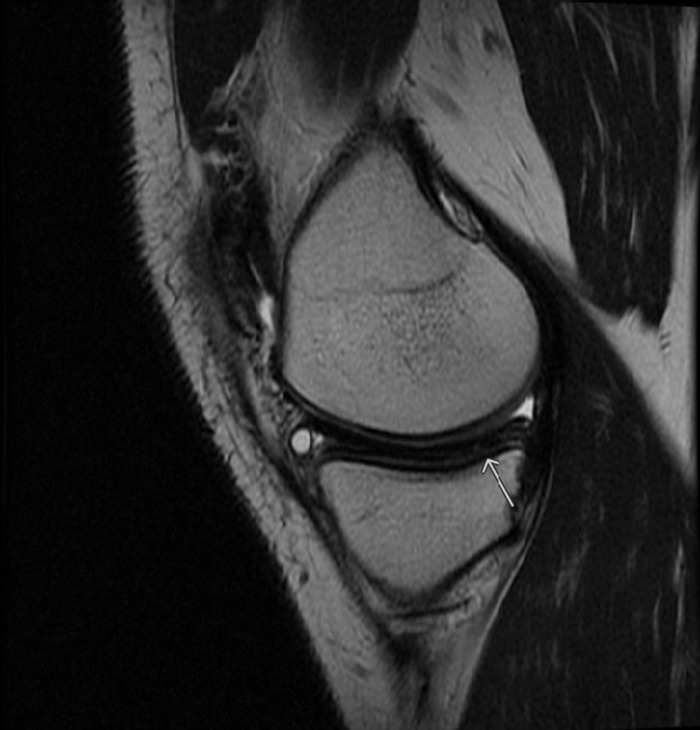
**Sagittal T2 magnetic resonance image shows a horizontal oblique undersurface tear of the posterior horn of the medial meniscus (arrow).**

Physical examination of the left knee showed medial joint line tenderness. Range of motion was measured from 0° to 130°. Lachman and posterior drawer tests were negative. The left knee was stable to both varus and valgus stresses with no locking or catching on McMurray testing. Left knee radiographs were unremarkable, but magnetic resonance imaging (MRI) confirmed discoid medial meniscus (Figure 1) and a horizontal oblique tear of the posterior horn of the medial meniscus (Figure 2).

**Figure 3.**
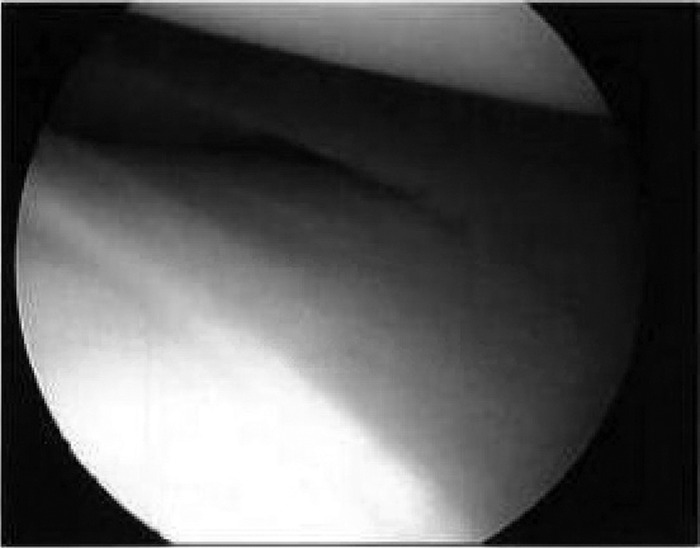
**Intraoperative arthroscopic view of the discoid medial meniscus.**

**Figure 4.**
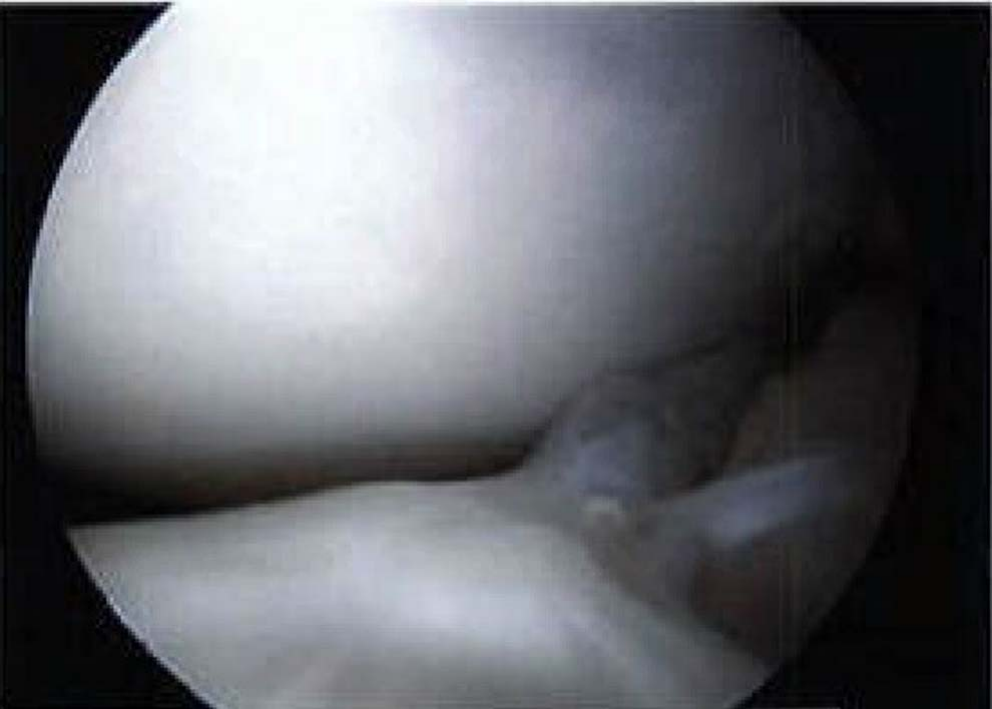
**Intraoperative arthroscopic view of the saucerized rim of the discoid medial meniscus.**

The patient underwent saucerization of the left discoid medial meniscus and medial meniscus repair. Lateral and medial parapatellar arthroscopic portals were used for intraoperative access. The anterior cruciate ligament (ACL) and lateral meniscus were intact. The discoid medial meniscus was visualized (Figure 3) and then saucerized with a 5-mm rim remaining (Figure 4). The meniscus tear was repaired using a Sequent Meniscal Repair Device (ConMed Linvatec) and 3 suture anchors. The patient tolerated the surgery well and was discharged on postoperative day 1 in a knee immobilizer with instructions for partial weight-bearing for up to 6 weeks and physical therapy starting 1 week after surgery for range of motion and quadriceps strengthening. The patient had full range of motion and good quadriceps function by 6 weeks postoperation, with eventual full return to physical activities. At 6-month follow-up, the patient had no mechanical symptoms or knee pain. Postoperative improvement continued unchanged at 1-year follow-up.

## DISCUSSION

The morphology and biomechanics of the medial tibiofemoral joint surface make the discoid medial meniscus unsuitable for load bearing.^6^ The fixed positioning of the medial meniscus to the medial tibial plateau predisposes the discoid meniscus to tearing in the setting of low impact trauma. Trauma is identified as the inciting cause of symptoms in 38% to 66% of patients with discoid medial meniscus.^3^ Pathophysiologic progression resulting from repetitive low-impact motion was inferred in our patient, as he had no history of acute trauma to the left lower extremity in the presence of a physically active lifestyle. For patients with refractory symptoms of pain and locking that progress to knee instability, surgical intervention has been shown to be the most effective method for symptom resolution compared to nonoperative measures such as physical therapy and corticosteroid injections.^14^ These findings were corroborated in our patient, as all pain and locking had resolved at 6-month follow-up.

A discoid meniscus can be diagnosed on sagittal MRI showing continuity between the anterior and posterior horns of the meniscus in 3 consecutive cuts. Further confirmation can be obtained with coronal images showing a transverse meniscal diameter >15 mm or involvement of >20% of the tibial width.^4^

While descriptive classification is used to characterize the grade, location, and size of standard meniscal injuries, no classification scheme exists for discoid medial meniscal injury. Variants have been described based on anterior horn insertion: (1) normal, (2) discoid meniscus with deficient insertion of the anterior horn onto the tibia with continuity of the anterior horn and anterior intermeniscal ligament over the ACL, and (3) discoid meniscus with the anterior horn in continuity with the ACL.^15^

Surgical management is indicated for symptomatic discoid medial meniscus tears. The saucerization is performed arthroscopically to prevent progressive degeneration of articular cartilage. Surgical approaches to repair discoid lateral meniscus tears have been well described.^16-18^ These techniques, however, are not universally applicable for repairing discoid medial meniscal tears. The medial meniscus is C-shaped as opposed to the uniform circular shape of the lateral meniscus.^1^ The medial meniscus has a posterior horn that is markedly wider than the anterior horn. The wide posterior horn of the medial meniscus and the large anteroposterior dimension compared to the lateral meniscus make visualization insufficient with the techniques used for discoid lateral meniscus repair.^19^

A literature review yielded 3 surgical approaches in addition to the standard 2-portal approach for treating symptomatic discoid medial meniscus tears.^10,19,20^ The standard 2-portal approach uses an anterolateral viewing portal and anteromedial working portal and is considered the gold standard for diagnostic knee arthroscopy but can also be used to treat discoid medial meniscus using the well-characterized inside-out technique.^21,22^ Treatment of posterior horn tears in a discoid medial meniscus using the 2-portal approach requires a larger valgus stress to extend the medial compartment compared to visualization of a discoid lateral meniscus, increasing the risk of iatrogenic medial collateral ligament injury.

Song et al described a 4-portal technique for convenient saucerization without risking anterior cartilage damage.^10^ Their additional high far lateral and low anteromedial portals allowed easy determination of remnant margins after saucerization. This 4-portal approach can be ideal for anterior horn tears of the discoid medial meniscus; however, visualization of the posterior horn is limited.

Wang et al introduced a technique that uses a central transpatellar tendon portal, a high anterolateral portal, and the standard anteromedial portal to access the discoid medial meniscus.^19^ The modified portals allow for better visualization of the posterior horn and provide a larger working space while eliminating blind spots that can occur when using the standard 2-portal approach. The Wang et al 3-portal approach has been shown to decrease operating time while restoring the anatomic morphology of the inner posterior rim of the medial meniscus.^19^ Limitations include short-term postoperative anterior knee pain caused by the central transpatellar tendon portal.

Kim et al also described a 3-portal approach that saves time, is less aggressive than the 4-portal technique, and excises the discoid medial meniscus in 1 piece.^20^ A limitation to using the high anterolateral portal for excision is the confined working space because of obstruction by the ACL and tibial intercondylar eminence. Removal of the posterior horn of the discoid medial meniscus can be difficult and increases the risk of iatrogenic injury to the ACL and anterior cartilage.

## CONCLUSION

Discoid medial meniscus is a rare congenital anatomic variant that predisposes individuals to meniscal tears. Symptomatic tears should be treated operatively with arthroscopic saucerization. The surgical approach should be carefully undertaken with adequate preoperative planning based on the location of the tear. Knowing the benefits and limitations of the available surgical approaches can help to optimize patient outcomes while minimizing risk of iatrogenic injury.

## Notes

This article meets the Accreditation Council for Graduate Medical Education and the American Board of Medical Specialties Maintenance of Certification competencies for Patient Care and Medical Knowledge.
